# UK adults’ implicit and explicit attitudes towards obesity: a cross-sectional study

**DOI:** 10.1186/s40608-015-0064-2

**Published:** 2015-09-04

**Authors:** Stuart W. Flint, Joanne Hudson, David Lavallee

**Affiliations:** Academy of Sport and Physical Activity, Faculty of Health and Wellbeing, Sheffield Hallam University, Sheffield, UK; Centre for Sport and Exercise Science, Sheffield Hallam University, Sheffield, UK; Leeds Beckett University, Room 103, Fairfax Hall, Headingly Campus, Leeds, LS6 3QS UK; School of Sport, University of Stirling, Stirling, UK

**Keywords:** Anti-fat attitudes, Implicit and explicit attitudes, Obesity

## Abstract

**Background:**

Anti-fat attitudes may lead to stigmatisation of and lowered self-esteem in obese people. Examining anti-fat attitudes is warranted given that there is an association with anti-fat behaviours. Previous studies, mainly outside the UK, have demonstrated that anti-fat attitudes are increasing over time.

**Methods:**

The study was cross-sectional with a sample of 2380 participants (74.2 % female; aged 18–65 years). In an online survey participants reported demographic characteristics and completed a range of implicit and explicit measures of obesity related attitudes.

**Results:**

Perceptions of obesity were more negative than reported in previously. Main effects indicated more negative perceptions in males, younger respondents and more frequent exercisers. Attitudes about obesity differed in relation to weight category, and in general were more positive in obese than non-obese respondents.

**Conclusions:**

This is the first study to demonstrate anti-fat attitudes across different sections of the UK population. As such, this study provides the first indication of the prevalence of anti-fat attitudes in UK adults. Interventions to modify these attitudes could target specific groups of individuals with more negative perceptions as identified here. Future work would be useful that increases understanding of both implicit and explicit attitudes towards obesity.

## Introduction

Over the past 20 years the number of people classified as overweight and obese has increased [1]. Alongside the more obvious health and economic implications is a less obvious and potentially significant societal impact: the stigmatisation of obese people and the development of anti-fat attitudes. Indeed stigmatisation and discrimination of obese people has increased in parallel with obesity prevalence [2, 3]. As might be expected, those who report anti-fat attitudes have a greater likelihood of stigmatising obese people which may occur in various settings [4–6]. It is suggested, for instance, that obese people are discriminated against in recruitment and promotion at work [5]. The increasing evidence for anti-fat attitudes presents considerable cause for concern as stigmatisation can result in elevated depression, general psychiatric symptoms, body image disturbance and lower self-esteem in obese people [7].

Research evidence for the prevalence of anti-fat attitudes comes mainly from the US [3] which might be expected as 68.8 % of adults are classed as overweight or obese, 35.7 % are obese and 6.3 % are morbidly obese [8]. However, obesity prevalence in the UK has increased and closely matches that observed in the US. In 2010, 42 % of males and 32 % of females are overweight and 26 % of all adults are classified as obese in England [9]. To date, studies of anti-fat attitudes in the UK have drawn small samples from narrow sections of the population, for instance exercise professionals [6]. Furthermore, the increase in overweight and obesity prevalence may have led to a normalisation process where overweight and obesity are viewed as the norm, resulting in less anti-fat attitudes over time. Alternately, greater exposure to overweight and obese people due to the increased prevalence may have led to greater anti-fat attitudes in the current UK population compared with previous years. Current UK Government policy relating to obesity fails to acknowledge the impact of obesity stigma and discrimination [10], yet research has identified that obesity stigma might hinder efforts to reduce obesity. Thus a more comprehensive investigation of anti-fat attitudes within the UK population that examines the impact of specific demographic factors is both timely and relevant. Research examining anti-fat attitudes in the UK population could provide pivotal information for policy makers and practitioners by directing anti-fat attitude interventions.

Research has identified that anti-fat attitudes differ in relation to individual characteristics including gender, age, exercise frequency and body mass index (BMI). In adult populations, respondents who are male, younger, exercise frequently and have a lower BMI are likely to report higher anti-fat attitudes [6, 11–13]. Internalisation occurs largely at an implicit level. Thus in addition to employing explicit measures of obesity attitudes, implicit measures may prove informative in this line of research and may negate limitations associated with explicit measures [14, 15].

Contemporary reports in the media depicting anti-fat attitudes, obesity stigmatisation and discrimination in the UK have increased over time; however, there is a paucity of empirical evidence to support these suggestions. This lack of evidence alongside previous research reporting detrimental links between ant-fat attitudes and behaviour with poorer body image and lowered self-esteem [7], suggests that examining obesity attitudes in the UK population is warranted. Thus, the present study aimed to examine anti-fat attitudes in a sample of UK adults (England, Ireland, Northern Ireland, Scotland, and Wales) and to compare attitudes in relation to gender, age, BMI and exercise frequency. UK adults were expected to report both implicit and explicit anti-fat attitudes (hypothesis 1). Higher levels of anti-fat attitudes were expected in males, younger participants, and more frequent exercisers (hypothesis 2).

## Methods

### Participants

Participants were 2380 volunteers (613 men, 1767 women; 18–65 years, mean age = 27.71, *SD* = 1.03 years) who were UK residents (confirmed in responses from England, Ireland, Northern Ireland, Scotland, and Wales) and predominantly white (93 %).

### Design and measures

This cross-sectional study was conducted online with data collection carried out over the course of a year. Participants reported their gender, age, height, weight, exercise frequency (hours per week) and perceptions of the words ‘*fat*’ (Q1: How insulting do you believe the word “fat” is?) and ‘*obese*’ (Q2: How insulting do you believe the word “obese” is?). To respond to Q1 and Q2 they used a 0-10 response scale, anchored by 0 = not at all and 10 = extremely insulting. BMI was calculated as weight (kg)/height (m)^2^ and individuals were assigned to the categories underweight (<18.5), normal weight (18.5–24.9), overweight (25–29.9) and obese (≥30; see Tables 1 & 2).

**Table 1:** Explicit attitudes towards obesity for gender, age, BMI and exercise frequency (mean and standard deviation) among UK adults aged 18–65 years in 2009–2010

Measure			Gender		Age (years)				BMI (kg/m^2^)			Exercise Frequency (hours per week)		
*n*		Male	Female	18–25	26–35	36–50	51–65	<18.5	18.5–24.9	25–29.9	≥30	0–3	4–7	8+
		*613*	*1767*	*1374*	*542*	*329*	*135*	*166*	*1518*	*440*	*256*	*1019*	*943*	*418*
ATOP	64.10	61.10	65.14	63.15	65.39	66.34	63.12	63.33	63.85	64.64	65.17	64.75	64.24	62.22
	(15.53)	(16.53)	(15.03)	(15.50)	(15.12)	(15.68)	(16.24)	(16.19)	(15.40)	(15.04)	(16.64)	(15.15)	(15.69)	(15.95)
BAOP	14.65	15.12	14.48	14.15	14.96	16.17	14.73	16.08	14.08	14.84	16.75	15.06	14.37	14.25
	(6.65)	(6.70)	(6.62)	(6.20)	(6.76)	(7.77)	(7.03)	(7.50)	(6.27)	(6.65)	(7.64)	(6.76)	(6.62)	(6.39)
AFAS	15.39	16.73	14.93	15.87	14.78	14.41	15.33	15.27	15.97	14.86	12.93	14.85	15.59	16.27
	(4.37)	(4.46)	(4.24)	(4.38)	(4.21)	(4.34)	(4.36)	(4.78)	(4.21)	(4.23)	(4.31)	(4.15)	(4.32)	(4.83)
F-Scale	3.74	3.80	3.72	3.77	3.70	3.67	3.76	3.72	3.79	3.71	3.55	3.71	3.77	3.77
	(0.47)	(0.50)	(0.47)	(0.48)	(0.45)	(0.48)	(0.49)	(0.48)	(0.46)	(0.46)	(0.51)	(0.46)	(0.47)	(0.52)

*ATOP, BAOP* Attitudes About Obese Persons Scale and Beliefs About Obese Persons Scale, *AFAS* Anti-Fat Attitudes Scale, *F-Scale* The Fat Phobia Scale short form

**Table 2:** Weight-related terms and implicit attitudes towards obesity for gender, age, BMI and exercise frequency (mean and standard deviation) among UK adults aged 18–65 years in 2009–2010

Measure			Gender		Age (years)				BMI (kg/m^2^)			Exercise Frequency (hours per week)		
*n*		Male	Female	18–25	26–35	36–50	51–65	<18.5	18.5–24.9	25–29.9	≥30	0–3	4–7	8+
		*613*	*1767*	*1374*	*542*	*329*	*135*	*166*	*1518*	*440*	*256*	*1019*	*943*	*418*
Q1:Fat	6.87	6.2	7.1	7.1	6.7	6.4	6.2	6.8	6.9	6.8	6.9	6.9	6.9	6.6
	(2.16)	(2.2)	(2.1)	(2.0)	(2.2)	(2.3)	(2.5)	(2.4)	(2.1)	(2.2)	(2.2)	(2.1)	(2.1)	2.3)
Q2:Obese	6.83	6.4	7.0	7.1	6.5	6.6	6.4	6.7	6.8	6.8	7.1	6.9	6.8	6.7
	(2.57)	(2.6)	(2.6)	(2.5)	(2.6)	(2.7)	(2.7)	(2.5)	(2.6)	(2.6)	(2.6)	(2.6)	(2.5)	(2.7)
*n*		491	1467	1198	442	231	87	140	1281	352	184	840	787	331
IAT D Score	147.81	161.08	143.37	130.40	107.76	269.88	266.94	178.74	147.57	170.70	83.99	157.31	144.26	132.16
	(691.65)	(702.51)	(688.16)	(714.80)	(656.51)	(643.07)	(626.36)	(685.82)	(696.66)	(683.66)	(677.85)	(672.02)	(704.30)	(711.88)

Q1: How insulting do you believe the word “fat” is?; Q2: How insulting do you believe the word “obese” is?; IAT: Implicit Association Test

Participants completed online versions of the Attitudes Towards Obese Persons and Beliefs About Obese Persons scales (ATOP, BAOP) [16] that measure both positive and negative attitudes towards obese persons and perceived controllability of obesity, respectively. Previous research [17] has suggested that those who perceive obesity to be controllable are more likely to have anti-fat attitudes. ATOP scores range from 0-120 across 20 items, where low scores represent more negative attitudes. BAOP scores range from 0-48 across 8 items, where low scores represent a stronger belief that obesity is controllable.

Participants also completed the Anti-Fat Attitudes Scale (AFAS) [18] that measures the magnitude of anti-fat attitudes via 5 items (scores range from 0 to 25 where higher scores represent stronger anti-fat attitudes), the 14 item F-Scale (Fat Phobia Scale short form) [19] that measures the degree to which individuals associate stereotypical characteristics with being fat (responses range from 0 to 5 where higher scores represent a perception that characteristics are associated with being fat), and the Implicit Association Test (IAT) [20] which was the only implicit measure used. The stimuli for in this computer-based measure of implicit attitudes towards fatness and thinness was previously used by Vartanian et al. [21]. The IAT does not directly measure attitudes but provides an indication of an implicit preference for fatness or thinness. Participants are presented with weight-related words and associate these as quickly as possible with different grouping categories as detailed below. In line with Lane et al. [22] the seven step procedure was employed, where participants respond to each of the following grouping categories: (1) *pleasant* or *unpleasant*; (2) *fat* or *thin*; (3) *fat/pleasant* or *thin/unpleasant*; (4) *fat/pleasant* or *thin/unpleasant* (stage 3 repeated); (5) *thin* or *fat*; (6) *fat/unpleasant* or *thin/pleasant*; and (7) *fat/unpleasant* or *thin/pleasant* (stage 6 repeated). Only steps 3, 4, 6 and 7 are used to measure implicit attitudes; the remaining steps were practice stimuli to engage participants with the process. Participants associated the words that appeared in the middle of the screen with either of the grouping category in the top left or top right of the screen using the E or I keys, respectively (e.g. for happy *pleasant* is located in the top left and *unpleasant* in the top right). Response latency to different pairs of grouping categories is measured in milliseconds (msec). Positive scores represent stronger anti-fat or pro-thin bias.

All measures except the IAT are explicit measures and employ likert-type scales. Higher scores on the AFAS, F-Scale, Q1 and Q2 and lower scores on the ATOP and BAOP represent more negative attitudes. Previous research has used the scales employed in the current study with different adult population groups reporting good reliability and validity (ATOP: [13]; α = .76; BAOP: [13]; α = .82; AFAS: [18]; α = .80; F-Scale: [19]; α = .87).

### Procedures

Ethical approval was obtained from Aberystwyth University Research Ethics Committee, UK, and potential participants were approached via 3 means of recruitment: (i) letters and emails distributed to UK businesses, councils, universities and higher education institutions (ii) social networking websites and (iii) conferences. Recruitment attempts were strategic to sample participants from as many counties across the UK as possible. Participants were asked to complete an online survey on attitudes and beliefs about obesity (as described above). Prior to completing all measures, participants were provided with information about the study and consented to participate. Measures were presented in counterbalanced order across participants to minimise order effects. No incentive was offered for participating in the study.

### Analysis

Total or mean scores were calculated for all measures and used in the analyses except the IAT where IAT D scores were calculated representing the difference between total response latency for the pairings *fat/pleasant* and *thin/unpleasant* versus *fat/unpleasant* and *thin/pleasant*. IAT D scores were calculated as recommended by Greenwald et al. [23]: (1) delete responses greater than 10,000 msec; (2) delete participants’ data where more than 10 % of responses have a response latency less than 300 msec; (3) compute the inclusive standard deviation for all responses in steps 3 and 6 and similarly in 4 and 7; (4) compute the mean latency for responses in steps 3, 4, 6 and 7; (5) compute the main differences (mean step 6 - mean step 3, and, mean step 7 - mean step 4); (6) divide each difference score by its associated inclusive standard deviation; and (7) calculate the D score as the equal weight mean of the two resulting ratios. D-scores range from -1000 to 1000 msec with positive scores indicative of anti-fat attitudes or pro-thin bias.

Mean scores reported in previous research that have employed the explicit anti-fat attitude measures of this study were used to determine if current data are indicative of anti-fat attitudes, as no criteria exist for interpreting these scores. Thus, the mean scores reported previously that were claimed to demonstrate anti-fat attitudes were used for comparison as follows: 59.7 and 17.9, ATOP and BAOP respectively [16]; 3.03, AFAS [18]; and 3.6, F-Scale [19].

Study hypotheses were examined by a series of Multivariate Analyses of Variance (MANOVA) conducted on the data for each independent variable (gender, age, BMI, exercise frequency) with all attitude measures as dependent variables (see Tables 1 & 2). gender had two levels; age had four levels as did BMI in line with the World Health Organisation BMI categories [24], exercise frequency had three levels in line with recommended UK physical activity guidelines representing: below recommended (0–3 hours per week), recommended (4–7 hours per week) and above recommended levels (8+ hours per week; see Tables 1 & 2). Follow-up one way ANOVAs for each independent variable were employed with Welch correction to examine multivariate effects (except for gender where an independent t-test was used). Post-hoc tests with Scheffé correction were used to follow-up significant ANOVA effects. One way ANOVAs were used to compare IAT D scores across different levels of independent variables. For all analyses α was set at .05.

## Results

Tables 1 and 2 present the descriptive statistics for all variables in relation to demographic and behavioural groups. Cronbach’s α were satisfactory for all scales: for the ATOP (.8), BAOP (.7), AFAS (.8), and F-Scale (.8). Table 3 reports significant overall univariate effects with results of follow-up tests to explore these discussed below.

**Table 3:** Results of one-way ANOVAs (*F*-statistics) for gender, age, BMI and exercise frequency among UK adults aged 18-65 years in 2009-2010

*d.f., total d.f.*	Age (years)	BMI (kg/m^2^)	Exercise Frequency (hours per week)
	(3, 2376)	(3, 2376)	(2, 2377)
ATOP	5.46**	.85	4.01*
BAOP	7.29**^a^	12.51**^c^	3.58*
AFAS	14.74**	39.72**^d^	16.40**^f^
F-SCALE	5.51**	20.34**	3.93*^g^
Q1:Fat	16.06**^b^	.28^e^	2.82^h^
Q2:Obese	8.39**	1.29	1.36

*BMI* body mass index, *d.f.* degrees of freedom, *ATOP*, *BAOP* Attitudes About Obese Persons Scale and Beliefs About Obese Persons Scale, *AFAS* Anti-Fat Attitudes Scale, *F-Scale* The Fat Phobia Scale short form; Q1: How insulting do you believe the word “fat” is?; Q2: How insulting do you believe the word “obese” is?; * *P* < .05; **P < .01; *d.f., total d.f.*: ^a^3, 477.5; ^b^3, 476.4; ^c^3, 482.2; ^d^2, 1118; ^e^3, 492.0; ^f^2, 1092; ^g^2, 1096; ^h^3, 493.5

The IAT D score (D = 147.8) indicated that, as anticipated, there was an overall anti-fat or pro-thin bias in the sample. Similarly, based on the criteria identified above, mean scores on explicit measures indicate negative attitudes towards obesity (see Table 1).

The MANOVA demonstrated main effects in relation to gender (*F*(6, 2373) = 38.22, *P* < .01), age (*F*(18, 6707) = 6.59, *P* < .01), exercise frequency (*F*(12, 4.07 = 4.19, *P* < .01) and BMI (*F*(18, 6707) = 11.07, *P* < .01). All dependent variables contributed significantly (*P* < .05) to these main effects with the exception of Q2 for exercise frequency and ATOP, Q1 and Q2 for BMI. The results of follow-up ANOVAs are detailed in Table 3, indicating significant age differences for all dependent variables. All variables except Q1 and Q2 differed in relation to exercise frequency, and, significant differences were observed for all variables except ATOP, Q1 and Q2 in relation to BMI. Post hoc test results are discussed below. The follow-up tests on the gender main effect indicated significant differences on all variables (see below).

### Gender

Males reported more negative attitudes towards obese people (ATOP), greater anti-fat attitudes (AFAS) and greater fat phobia (F-Scale) than females (*t*(985.25) = -5.34, *P* < .01; *t*(2378) = 8.92, *P* < .01; *t*(2378) = 3.41, *P* < .01, respectively). In contrast, females reported stronger beliefs that obesity is controllable (BAOP: *t*(2378) = 2.05, *P* < .05) and perceived the words fat (Q1: *t*(1022) = -9.18, *P* < .01) and obese (Q2: *t*(2378) = -5.10, *P* < .01) as more insulting.

### Age

Eighteen to twenty five year olds reported more negative attitudes towards obese people (ATOP; *P* <. 01), greater anti-fat attitudes (AFAS; *P* < .01) and greater fat phobia (F-Scale; *P* < .01) than 26–50 year olds. 18–25 year olds also reported stronger beliefs that obesity is controllable (BAOP; *P* < .01) than 36–50 year olds, and, perceived the words fat (Q1) and obese (Q2) as more insulting than 26–35 year olds (*P* < .01), 36–50 year olds (*P* < .01) and 51–65 year olds (*P* < .01).

### Exercise frequency

Participants who exercise 8 or more hours a week reported more negative attitudes towards obese people (ATOP; *P* < .01) and greater anti-fat attitudes (AFAS; *P* < .01) than those who exercise 0–3 hours a week. They also reported greater anti-fat attitudes (AFAS) than those who exercise 4–7 hours a week (*P* < .01), who in turn reported greater anti-fat attitudes (AFAS; *P* < .01) and fat phobia (F-Scale; *P* < .01) than those who exercise 0-3 hours a week. Overall, the explicit results demonstrate that males, younger respondents and more frequent exercisers reported more negative perceptions of obesity.

### BMI

Anti-fat attitudes (AFAS) were greater in underweight and overweight than obese participants (*P* < .01) and in normal weight compared with overweight and obese participants (*P* < .01). Fat phobia (F-Scale) was lower in obese than underweight, normal weight and overweight participants (*P* < .01), and in overweight compared with normal weight participants (*P* < .01). Normal weight participants believed that obesity is more controllable (BAOP) than underweight and obese participants (*P* < .01), as did overweight compared with obese participants (*P* < .01).

### Correlations between explicit measures

A number of correlations were evident between explicit measures (see Table 4). A positive correlation between ATOP and BAOP scores was observed, where more negative attitudes towards obese persons were associated with a stronger belief that obesity is controllable. A positive correlation between AFAS and F-Scale scores was also evident, where more anti-fat attitudes were associated with greater fat phobia. Other positive correlations were evident between BAOP and Q2, Q1 and Q2, and Q2 and F-Scale scores. This suggests that perceptions that the word obese is more insulting were associated with stronger beliefs that obesity is controllable, perceptions that the word fat is more insulting and greater fat phobia.

**Table 4:** Correlations between the explicit measures

	ATOP	BAOP	AFAS	F-SCALE	Q2	Q3
ATOP		.43^a^	-.59^a^	-.58^a^	-.04	-.07^a^
BAOP			-.47^a^	-.05^b^	-.06^a^	.53^a^
AFAS				.62^a^	-.04	.03
F-SCALE					.02	.11^a^
Q1						.36^a^
Q2						

*ATOP*, *BAOP* Attitudes About Obese Persons Scale and Beliefs About Obese Persons Scale, *AFAS* Anti-Fat Attitudes Scale, *F-Scale* The Fat Phobia Scale short form; Q1: How insulting do you believe the word “fat” is?; Q2: How insulting do you believe the word “obese” is?; ^a^Correlation is significant at the .01 level; ^b^Correlation is significant at the .05 level

A negative correlation was evident between ATOP and AFAS scores, where more negative attitudes towards obese persons were associated with higher levels of anti-fat attitudes. A negative correlation also observed between BAOP and AFAS scores, where stronger beliefs that obesity is controllable were associated with more anti-fat attitudes. BAOP and F-Scale scores were negatively correlated indicating that stronger beliefs that obesity is controllable are associated with greater fat phobia. Finally, negative correlations were also found between scores on the ATOP and Q2, ATOP and F-Scale, and BAOP and Q2. This suggests that more negative attitudes towards obese persons are associated with perceptions that the word obese is more insulting and with greater fat phobia, and that stronger beliefs that obesity is controllable are associated with perceptions that the word obese is more insulting.

## Discussion

The study examined anti-fat attitudes in a cross-section of UK adults (England, Ireland, Northern Ireland, Scotland, and Wales) and compared attitudes in relation to gender, age, BMI and exercise frequency. Implicit and explicit anti-fat attitudes were evident in our sample of UK adults in line with hypothesis 1. Anti-fat attitudes were higher in males, younger participants and more frequent exercisers, in support of hypothesis 2.

Our findings illustrate that in UK adults, anti-fat attitudes appear to be widespread. Given the stigmatisation that can result from pervasive anti-fat attitudes, interventions to modify anti-fat attitudes are required. Anti-fat attitudes appear to be robust and have proven difficult to modify [25]; however some promise has been reported in altering beliefs about the causes of obesity [26]. Current study findings suggest that particular groups could be targeted with attitude modification interventions: males, younger individuals, and frequent exercisers. There are plausible explanations for greater anti-fat attitudes in all these groups: males tend to be less empathetic than females [27], a heightened awareness of body appearance in younger individuals, and, the incidence and possible acceptance of weight-related criticism in exercise environments [28–30]. These are all modifiable factors suggesting that interventions targeting these may well be successful. Our descriptive data does not offer support for the explanations we propose. Thus they require confirmation in future work before being used to underpin interventions to address negative perceptions of obesity in these groups. Nevertheless, given that anti-fat attitudes can lead to the stigmatisation of obese people [31]; our findings highlight the need for anti-fat attitude intervention with UK adults.

Our data reveal some interesting, although possibly contradictory, findings regarding perceptions of the controllability of obesity and of the descriptors fat and obese. Females and younger respondents tended to perceive obesity as more controllable and the labels fat and obese as more insulting than males and older respondents. For younger respondents this appears logical as they reported more anti-fat attitudes, thus they perceive labels associated with the condition as insulting. In addition, correlations from the current study that support previous research [32], suggest that these anti-fat attitudes are likely to derive partially from the belief that obesity is controllable and that obese people are responsible, indeed to blame, for their condition. This interpretation does not explain the same pattern seen in females as they did not report particularly strong anti-fat attitudes. Thus it may be that the participants perception of the labels used to describe obese people are not directly related to, or derived from, their evaluative perceptions of obese people themselves.

The differences observed in perceived controllability of obesity in relation to BMI are unclear. Obese respondents reported lower perceived controllability than normal and overweight respondents. This may serve as a self-protective mechanism in obese people to maintain self-esteem as they apportion less self-blame for their obesity [17]. Or, it may reflect their lived experience of being obese, as substantial evidence suggests a role for uncontrollable factors such as genetics in becoming obese [33], and, obese people are aware of their own exercise and nutrition habits, unlike external others. Less clear is the finding that perceived controllability was lower in underweight compared with normal weight respondents. Possibly underweight people recognise that weight at both extremes of the continuum is not always within the individual’s control if they themselves suffer from an eating disorder or are not underweight through choice. These explanations are of course highly speculative given that our study did not seek to identify explanations for different obesity attitudes. Whilst they intuitively make sense future research is clearly warranted to examine these suggestions.

Interestingly, despite the differences observed in the explicit measures, as discussed above, there was a null effect in relation to implicit attitudes when compared across the demographic factors. Current study findings demonstrate that UK adults have implicit anti-fat or pro-thin bias, but no differences were observed for almost all of the demographic factors. Previously it has been suggested that implicit measures counter some of the limitations of explicit measures, such as response bias and demand characteristics [14, 15]. Thus, differences observed in explicit responses, may have been a result of participants reducing the extent of their anti-fat attitudes, whilst this was not observed via implicit measures. Thus the current study findings highlight the need to examine both implicit and explicit attitudes towards obesity.

Regardless, our findings do underscore the importance noted previously of recognising the terms used to describe overweight and obesity [34]. Although medical professionals may use the term obese in an objective sense to describe a clinical condition, for our sample and in particular younger, female respondents, this was perceived as an insulting label. This finding reinforces previous suggestions that the term obese should be avoided [35]. Moreover the findings go beyond previous suggestions that have demonstrated that the term ‘obese’ should be avoided with obese patients, as our study demonstrates that the term is perceived as insulting in participants across BMI categories. Recently, guidelines have been developed for using language more sensitively to avoid objectification of the individual and placing the condition before the person, for instance the term ‘diabetic’ has been replaced by ‘people with diabetes’ [36]. Similar adjustments would seem appropriate when discussing obese people. Studies that compare perceptions of obese people when different labels are used to describe them would be simple to conduct but may produce illuminating findings to guide the somewhat complex issue of terminology use.

Both fat phobia and anti-fat attitudes tended to be lower in overweight and obese respondents in line with previous research [7]. We might therefore suggest that obesity stigmatisation comes from non-obese people, which may serve to further alienate obese people. Interestingly though, regardless of BMI, when measured implicitly, all respondents reported an anti-fat or pro-thin bias. Even if not expressed explicitly, it appears that obese people in our sample have internalised the same anti-fat or pro-thin attitudes as have non-obese people. These findings present less apparent contradiction when we consider that self-reported attitudes are open to manipulation by the respondent, whether consciously or not [15]. In this instance, this manipulation could have occurred because obese people felt uncomfortable publicly denigrating themselves in explicitly reporting their attitudes towards obese people. Similarly, females’ implicit attitudes did not differ from males’ in their anti-fat or pro-thin bias but they explicitly reported less negative perceptions of obesity. This may reflect the greater social desirability tendency in females [37], or, as suggested above, greater empathy in females. Clearly, future studies are needed that replicate the implicit measure used here to tease out these individuals’ ‘true’ responses.

Whilst the sampling strategy has limitations, the sample was successful in other ways. For example, the sample included respondents from every country across the UK and is the first study to obtain perceptions from a large group of participants from the UK. This was made possible due to the online sampling method that offers alternative benefits, for example, internet-based studies provide an opportunity to achieve a greater diversity in their samples [38]. These authors also argue that preconceptions about internet-based research are incorrect. For instance that the resultant sample will be younger, but the sample is often similar to that observed in traditional university based samples. They also note that there is no evidence that results of internet-based research are confounded by false data or repeat responders, nor do internet-based questionnaires diminish the psychological properties reported for pen-and-paper versions, both common preconceptions. Furthermore, whilst the sampling method means the researcher is not present during data collection, some respondents did make contact with the researcher to address queries.

We do however acknowledge that there are inherent biases to this approach, which may have resulted in the greater proportion of respondents who were white, middle class, more highly educated and of a higher social economic status. The majority of respondents were female (74.2 %), aged 18–25 years (57.7 %) and were students (47.2 %). As we might expect with a volunteer, opportunistic sample, our sample composition does not exactly match that of the UK population [39]. Despite attempts to sample a varied population, a more strategic sampling approach to ensure sub-groups were more equally represented might have strengthened the conclusions drawn from these data. Our sample composition does not match the demographic profile of the UK population [39], which impacts the generalizability of the data. Nevertheless, our findings reflect those obtained with similar population subgroups, such as more anti-fat attitudes in males [10]. Thus it is likely that if a ‘representative’ sample were examined, findings would be similar to those obtained here.

The reader should be aware of these limitations when considering our findings but given the paucity of current evidence from UK samples, we offer an initial contribution to stimulate further study. It is also important to highlight that the implicit measure we employed represents both a strength and a limitation of our study. Its strength lies in offering a measure of what some authors have described as ‘true’ attitudes [15] but given the format of Implicit Association Tests responses can only indicate anti-fat or pro-thin bias and not an absolute level of anti-fat attitude.

The current study is the first to comprehensively examine obesity attitudes in the UK population, demonstrating that UK adults report both implicit and explicit anti-fat attitudes. To date, obesity stigmatisation and discrimination is not included in UK health policy such as the Department of Health’s Obesity and Health Eating policy [10]. Based on the current study findings, we suggest that obesity stigmatisation and discrimination is incorporated into the policy as an action. This appears to be particularly relevant with previous research suggesting that obesity stigmatisation and discrimination may be a barrier to engaging in some of the actions that are already present such as physical activity [28, 40].

## Conclusions

The current study is the first to examine obesity attitudes across different sections of the UK population and in doing so highlight population groups with higher anti-fat attitudes. The present results extend the growing body of literature indicating that rising levels of obesity present challenges not only at an individual but also at a societal level, as anti-fat attitudes appear pervasive, albeit not to the same degree, across the different groups we sampled. A novel contribution of this study is that this is the first large scale examination of UK adults’ perceptions of obesity and how these differ between population groups.

This study is also the first to demonstrate that perceptions of obesity are similar to those reported in other countries, predominantly the US. Subsequently, the findings of our research call for anti-fat attitude intervention in the UK. Education about the uncontrollable causes of obesity can reduce anti-fat attitudes [25], and given that our study demonstrates strong beliefs that obesity is controllable in UK adults, future research should consider this when designing interventions for certain population groups. Building on present study findings, future research could examine the efficacy of interventions to modify both implicit and explicit anti-fat attitudes and identify explanations for differences in obesity perceptions in subgroups of the population.
